# Methodological Evaluation of Coda Wave Interferometry for Concrete Monitoring: Numerical Assessment of Mechanical Load, Temperature, and Moisture Coupling

**DOI:** 10.3390/ma19214463

**Published:** 2026-10-23

**Authors:** Gabriel Valla Traibel, Giao Vu, Erik H. Saenger

**Affiliations:** 1 Department of Mechatronics and Mechanical Engineering, Zentralcampus, Bochum University of Applied Sciences, Am Hochschulcampus 1, 44801 Bochum, Germany; 2 Fraunhofer Research Institution for Energy Infrastructures and Geothermal Systems, Am Hochschulcampus 1, 44801 Bochum, Germany; giao.vu@ieg.fraunhofer.de; 3 Department of Civil and Environmental Engineering, Zentralcampus, Bochum University of Applied Sciences, Am Hochschulcampus 1, 44801 Bochum, Germany; 4 Institute of Geology, Mineralogy and Geophysics, Ruhr University Bochum, Universitätsstraße 150, 44801 Bochum, Germany

**Keywords:** coda wave interferometry, concrete, numerical modelling, ultrasound, damage, temperature, moisture

## Abstract

Coda Wave Interferometry (CWI) is a highly sensitive ultrasonic technique capable of detecting subtle changes in heterogeneous materials such as concrete. Due to multiple scattering, the coda portion of the ultrasonic wavefield is particularly responsive to small variations in elastic properties, which may arise from mechanical loading or environmental effects. In concrete structures, changes in stress, temperature, and moisture content are known to influence ultrasonic velocities in a coupled manner, making the interpretation of monitoring data challenging. In this study, the sensitivity of CWI to mechanical load, temperature, and moisture variations in concrete is investigated through numerical simulations. Realistic numerical models of concrete are employed to simulate ultrasonic wave propagation under controlled perturbations representing mechanical loading and environmentally induced changes in material properties. Relative velocity variations, quantified as relative velocity change (dv/v), are extracted using CWI and analyzed to assess the individual and combined influence of the considered parameters. Specifically, model-based findings indicate that within the investigated numerical configuration: (i) relative humidity variations between 35% and 95%, modeled via quality factor *Q* changes alone, induce negligible relative velocity shifts (<0.08%) while increasing waveform decorrelation; (ii) temperature variations between 0 °C and 40 °C produce a linear dv/v response of approximately −0.0188%/K via mortar matrix softening; and (iii) uniaxial tensile damage exhibits a strongly nonlinear, regime-dependent dv/v signature characterized by a steep drop during diffuse microcracking (D<0.08) followed by a flatter response in the post-peak softening regime (D>0.2). Experimentally validated trends reported in the literature are used as reference to guide model calibration and interpretation, while all analyses are performed in a fully numerical framework. The results demonstrate that CWI captures distinct responses associated with load-induced and environmentally induced changes, and they highlight the potential and limitations of numerical modeling for disentangling mechanical and environmental effects in concrete monitoring applications under the specified assumptions.

## 1. Introduction

Concrete is a widely used construction material whose mechanical performance and durability are strongly affected by both loading conditions and environmental factors. Variations in stress, temperature, and moisture content can alter the elastic properties of concrete, influencing its stiffness, wave velocity, and damage evolution over time [1]. For structural health monitoring applications, it is therefore essential to employ diagnostic techniques capable of detecting small changes in material properties [2,3] while distinguishing between mechanical and environmental effects.

Ultrasonic testing methods have long been used for the evaluation of concrete structures. Among them, CWI [4] has gained increasing attention due to its exceptional sensitivity to weak perturbations in heterogeneous media. Unlike direct wave arrivals, coda waves result from multiple scattering events within the material [5], causing them to sample a large volume of the structure. As a consequence, even minor changes in elastic moduli, microcrack density, or boundary conditions can lead to measurable variations in the coda waveforms, typically expressed as relative velocity changes or waveform decorrelation [2].

Previous studies have demonstrated the capability of CWI to detect damage progression and stress-induced effects in concrete [2,6,7]. However, environmental influences such as temperature fluctuations and moisture variations are known to produce velocity changes of similar magnitude [6], potentially masking or mimicking mechanical effects. This sensitivity, while advantageous, poses a challenge for practical applications, as the interpretation of CWI results requires a clear understanding of how different parameters affect the coda wave response.

To define the research gap more precisely, while extensive experimental and numerical studies have evaluated individual environmental or mechanical influences on CWI responses [2,6,7], the potential multipysical coupling among moisture, temperature, attenuation, stiffness, and wave velocity in concrete remains challenging to isolate under field conditions. The existing literature often treats these factors independently or lacks systematic numerical frameworks capable of decoupling global velocity shifts, global attenuation changes, and local damage evolution. The present study advances existing approaches by establishing a controlled, fully numerical framework that explicitly differentiates these mechanisms under specific modeling assumptions.

A central working hypothesis of this study, supported by findings in the literature [6,8], is that the three governing parameters affect the coda wave signal through physically distinct mechanisms. Temperature changes are assumed to produce a global effect on wave velocity: as temperature alters the elastic moduli of the cement matrix throughout the specimen, the corresponding velocity shift is homogeneous across the entire propagation medium. Moisture changes, by contrast, are assumed to induce a global change in attenuation: variations in relative humidity modify the quality factor *Q* of the material, increasing energy dissipation as pore saturation grows, without significantly perturbing the wave speed (under the adopted attenuation-only assumption). Mechanical damage, on the other hand, produces a local change in elastic properties: crack initiation and propagation alter the stiffness distribution heterogeneously within the specimen, affecting the coda through localised scattering perturbations. This tripartite classification, global velocity shift (temperature), global attenuation change (moisture), and local stiffness change (damage), provides the conceptual basis for the numerical sensitivity analysis that follows.

The objective of this work is to investigate the influence of mechanical load, temperature, and moisture on coda wave signals in a concrete specimen using Coda Wave Interferometry. By systematically analyzing these effects, the study aims to quantify their respective contributions to relative velocity variations and to explore their implications for structural monitoring. The outcomes of this research provide valuable insights for the development of robust CWI-based monitoring strategies for concrete structures subjected to varying mechanical and environmental conditions.

## 2. Theoretical Background

### 2.1. Coda Wave Interferometry

Ultrasonic monitoring techniques have gained increasing interest due to their ability to detect small changes in materials and structures. Conventional ultrasonic methods typically focus on direct wave arrivals and parameters such as time of flight or wave velocity. While well established, their sensitivity is limited when changes are subtle or occur outside the direct propagation path between source and receiver [9].

**Figure 1:**
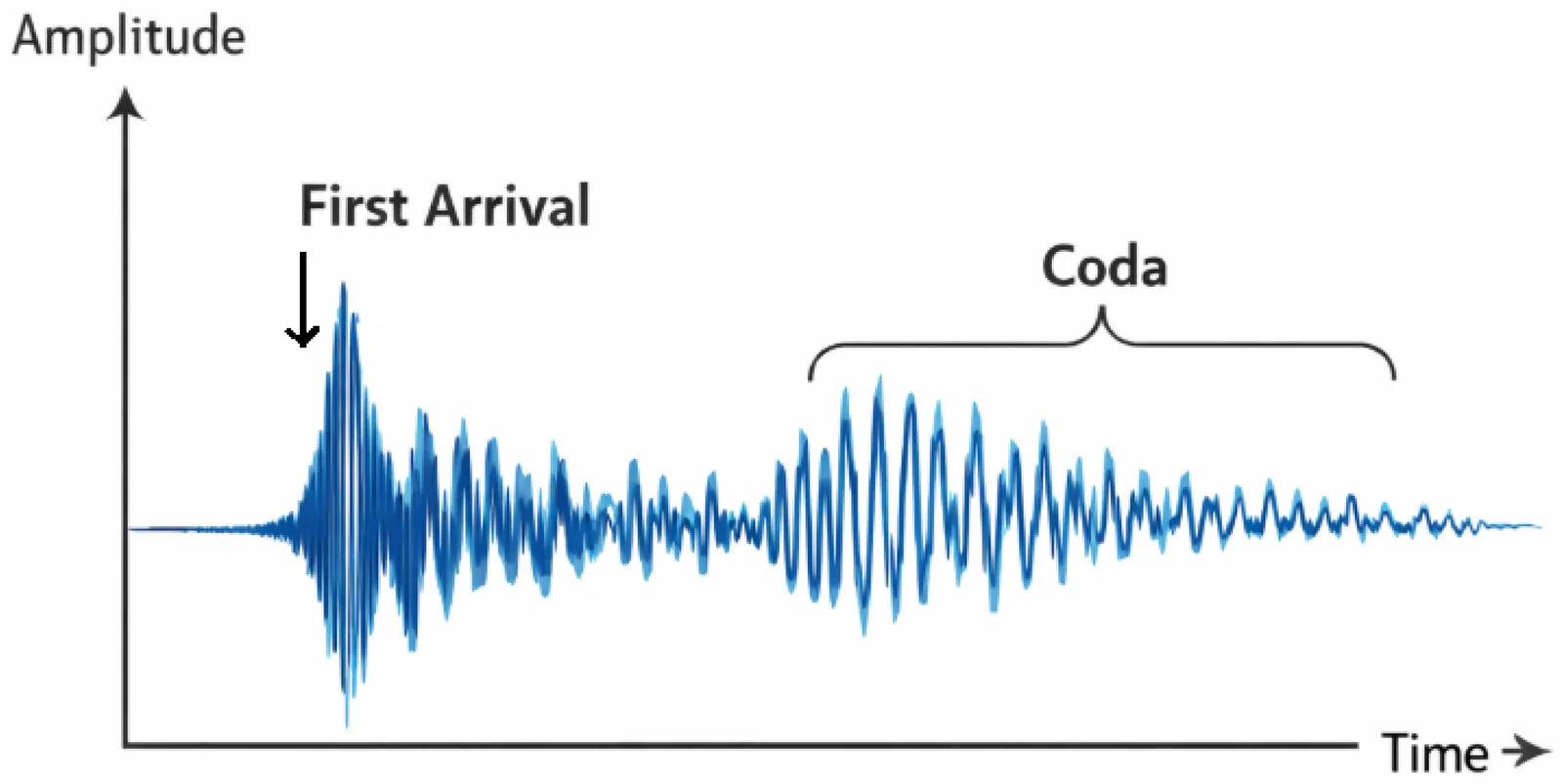
Schematic representation of the later part of the signal (coda).

CWI addresses these limitations by exploiting the later part of the ultrasonic signal, known as the coda, illustrated in Figure 1. Originally developed in seismology to monitor weak velocity variations in the Earth’s crust [10], CWI has since been successfully applied to engineering materials such as concrete, where strong heterogeneity leads to multiple scattering [9].

**Figure 2:**
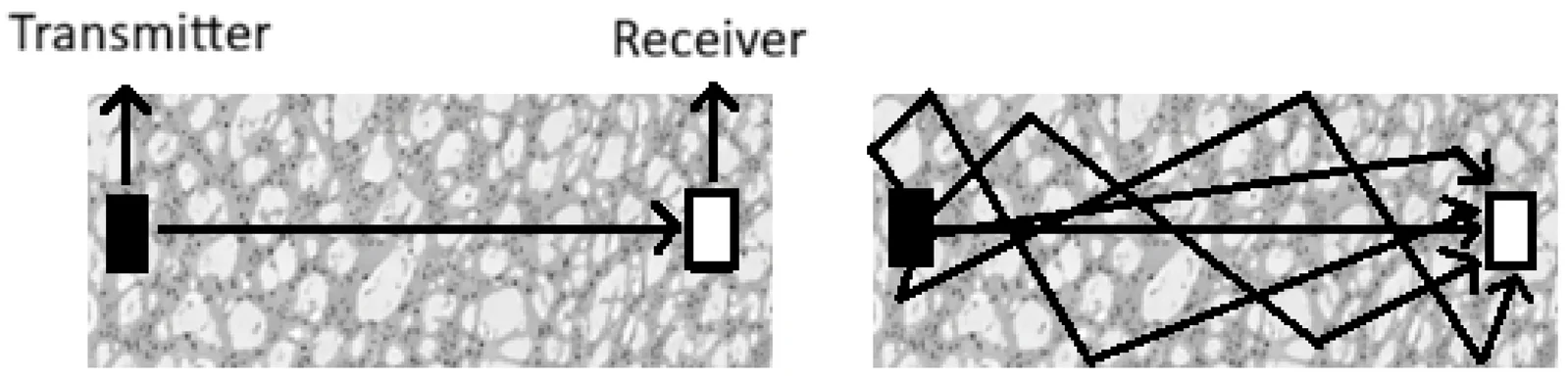
Schematic representation of the scattering effect of a wave propagating through concrete. The left side depicts the principle of time of flight (first arrival) measurements while the right side illustrates the ultrasonic wave travel paths involved in the coda. Adapted after [11].

In heterogeneous media, ultrasonic waves do not propagate along a single path but are scattered by aggregates, pores, grain boundaries, and cracks. After the initial direct arrival, the recorded signal becomes increasingly complex as waves undergo multiple reflections and scattering events as shown in Figure 2.

This late-time wavefield, referred to as the coda, results from the superposition of waves that have traveled along many different trajectories [5]. Because these paths are long and sample a large volume of the material, the coda is extremely sensitive to small changes in elastic properties or stress conditions [12].

The basic principle of CWI consists of comparing two ultrasonic signals recorded with the same source–receiver configuration at different states of the material. One signal serves as a reference (u(t)), while the second corresponds to a perturbed state ($\tilde{u}\left(t\right)$) caused by loading, temperature variation, moisture change, or damage [5]. If the material remains unchanged, the signals are nearly identical; otherwise, small phase shifts or time delays appear, particularly in the coda. These delays arise from cumulative travel-time changes along multiple scattering paths, making CWI significantly more sensitive than methods based solely on direct waves [4,10].

Prior to applying CWI, ultrasonic signals typically undergo preprocessing to remove offsets, electrical noise, and undesired frequency components. Bandpass filtering and careful selection of a coda time window are essential to ensure that waveform comparisons reflect material changes rather than measurement artifacts [5,9].

Quantitative analysis in CWI is commonly performed using cross-correlation, which measures the similarity between two signals as a function of time shift [4]. The stretching technique [5] is performed to obtain relative velocity changes (dv/v) in the structure. This consists of deriving a stretching factor ϵ=−dv/v when maximizing the cross-correlation coefficient CC(ϵ) between a reference signal u(t) and a stretched perturbed signal $\tilde{u}\left(t\left(1-\epsilon \right)\right)$. The relation used is expressed in Equation (1), where $t_{1}$ and $t_{2}$ define the time interval (coda window) in which the velocity change is derived:

(1) $$CC\left(\epsilon \right)=\frac{\int_{t_{1}}^{t_{2}}u\left(t\right)\tilde{u}\left(t\left(1-\epsilon \right)\right)dt}{\sqrt{\int_{t_{1}}^{t_{2}}u^{2}\left(t\right)dt\int_{t_{1}}^{t_{2}}{\tilde{u}}^{2}\left(t\left(1-\epsilon \right)\right)dt}}$$

### 2.2. Quality Factor and Attenuation

In wave propagation studies, attenuation and the quality factor *Q* are central descriptors of how wave energy diminishes in a material. Attenuation refers to the reduction in amplitude or energy of a wave as it travels through a medium. This reduction arises from anelastic energy loss due to internal friction, scattering, and other dissipative mechanisms in the material itself [13], in addition to geometric spreading (which redistributes energy across expanding wavefronts without removing total energy) [14].

The quality factor *Q* is a dimensionless parameter that characterizes the relative rate of energy loss, quantifying how efficiently a medium stores elastic energy compared to how much energy it loses per cycle of oscillation [13]. A high *Q* corresponds to weak intrinsic attenuation (slow energy decay), whereas a low *Q* indicates strong attenuation (rapid energy loss). The relationship between amplitude decay, frequency, and quality factor is formulated in Equation (2) through the amplitude A(ω,t) of a band-limited ultrasonic signal at angular frequency ω=2πf and time *t*:

(2) $$A\left(\omega ,t\right)=ct^{-a}\exp\left(-\frac{\omega t}{2Q}\right)$$

where *c* is a constant source amplitude, $t^{-a}$ accounts for geometric spreading (with a=1 assumed for body waves) [8], and the exponential term represents intrinsic attenuation governed by *Q*.

### 2.3. Numerical Considerations

Numerical simulation of elastic wave propagation is an essential tool for investigating ultrasonic phenomena in heterogeneous materials where analytical solutions are not feasible. Finite-difference (FD) methods are widely used due to their conceptual simplicity, flexibility, and computational efficiency, allowing for detailed modelling of scattering, reflections, and wave-mode conversions in complex media.

In standard staggered-grid schemes [15], different physical quantities are defined at distinct spatial locations within each grid cell, which enhances the accuracy of spatial derivatives and reduces numerical instabilities. However, classical staggered grids can suffer from numerical anisotropy, where wave velocities depend weakly on the direction of propagation relative to the grid axes, an effect that becomes more pronounced for coarse discretisations or high-frequency waves [16].

To mitigate these limitations, Ref. [16] proposed the rotated staggered grid, in which finite-difference operators are oriented along diagonal directions rather than solely along the Cartesian axes. Although the grid itself remains Cartesian, this rotation leads to more isotropic numerical behaviour and reduces grid-induced artefacts. Stress and velocity components are staggered diagonally with respect to each other, and spatial derivatives are approximated using combinations of diagonally adjacent grid points, resulting in a more balanced sampling of wave propagation in all directions. Due to these advantages, the rotated staggered-grid FD method is adopted in the present work to model ultrasonic wave propagation in concrete.

The accuracy of numerical predictions of transient structural responses depends not only on the spatial and temporal discretization but also on the formulation used to represent the governing equations and the quantities of interest. For example, Ref. [17] developed a modified orthonormal polynomial series-expansion method for the transient response of thin beams under slamming loads, demonstrating improved convergence and suppression of oscillations associated with the Gibbs phenomenon when calculating structural response quantities. Although their problem and numerical approach differ from the finite-difference wave-propagation framework employed here, their findings provide a complementary illustration of how numerical formulation can affect the accuracy of transient-response predictions.

## 3. Methodology

In this section, the methodological framework adopted to investigate the influence of environmental and mechanical factors on the coda wave response of concrete is established. The numerical modeling strategy, the definition of environmental loading scenarios, and the procedures used to introduce moisture variations, temperature changes, and internal damage are described. A dedicated section within this chapter details the numerical concrete model employed for wave propagation simulations. The selected ranges of relative humidity and temperature are intended to represent typical service conditions encountered by concrete structures in civil engineering applications, such as bridges and building components exposed to natural climatic variations. In service, concrete elements are commonly subjected to relative humidity levels ranging from moderately dry to near-saturated conditions, as well as to seasonal and daily temperature fluctuations around ambient values, depending on geographic location and exposure class. Experimental and field studies have shown that such environmental variations significantly affect the elastic properties and ultrasonic response of concrete without necessarily inducing irreversible damage, provided that extreme conditions are avoided [18]. Consequently, the ranges considered in this study are chosen to reflect realistic operational environments while ensuring consistency with previously reported experimental investigations on the environmental sensitivity of ultrasonic and coda wave measurements in concrete.

### 3.1. Concrete Model and Numerical Reproducibility

**Figure 3:**
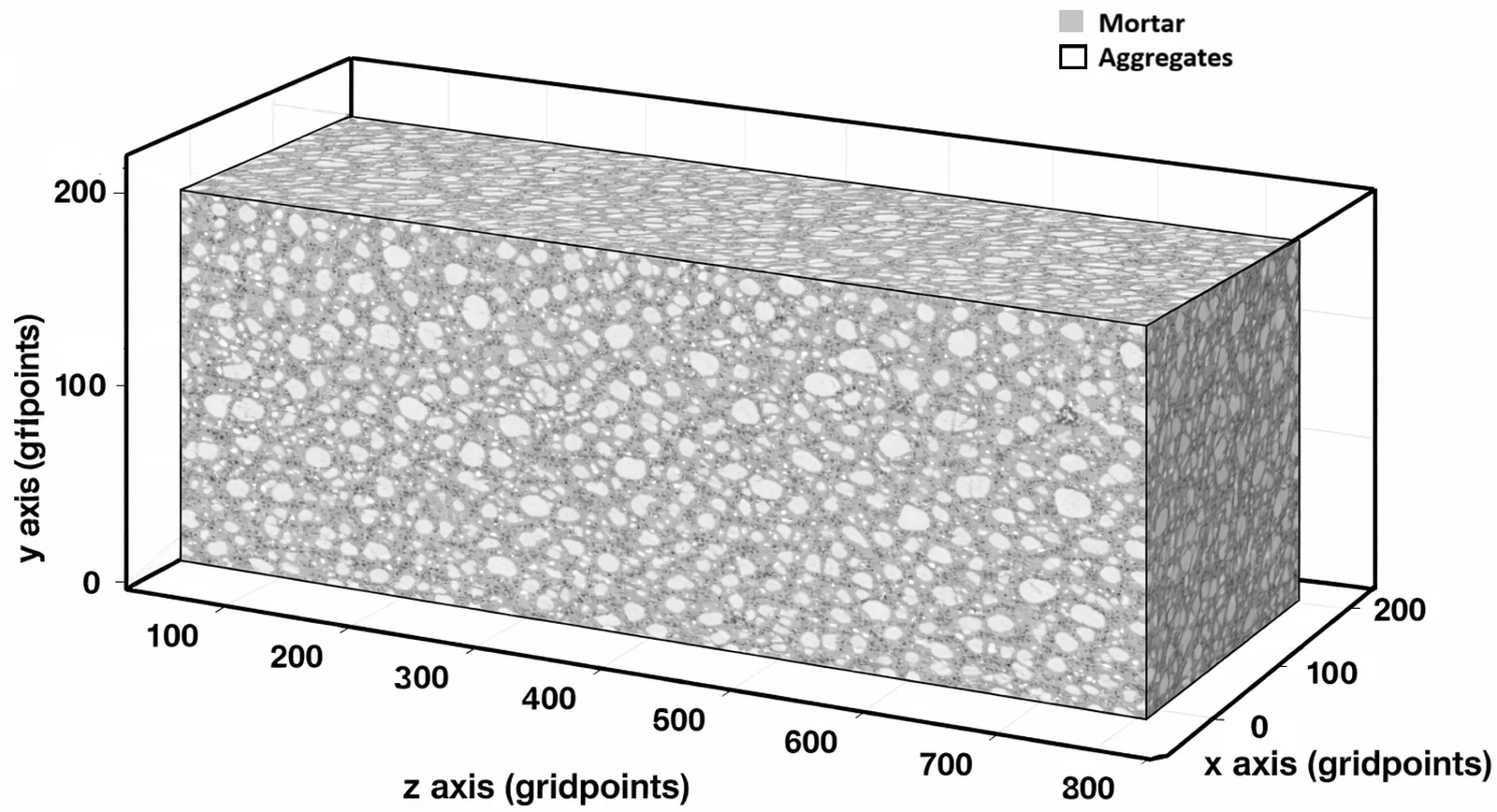
Elastic model of the concrete specimen. Axis labels are given in gridpoints with 1 gridpoint equal to 0.5 mm. Adapted from [19].

The numerical model follows the general concept of ultrasonic testing of concrete previously presented by [19]. The simulated concrete specimen has dimensions of $400\times 100\times 100\;{\mathrm{mm}}^{3}$. Applying a spatial resolution of Δx=0.5mm yields a computational grid consisting of 800 grid points in the longitudinal direction (*z*-axis) and 200 grid points in each transverse direction (*x*- and *y*-axes), as displayed in Figure 3.

The specimen is surrounded on all sides by a two-grid-point vacuum layer. Within this layer, wave velocities are set to zero and the density is assigned a small non-zero value ($\rho =0.0001\;{\mathrm{kg}/m}^{3}$), approximating a low-impedance exterior around the concrete sample. This is a simplified vacuum-padding treatment rather than a dedicated perfectly matched layer (PML) or other explicitly absorbing boundary condition; therefore, the possibility of residual boundary reflections cannot be excluded.

The generation of the heterogeneous concrete model incorporates aggregate distribution, volume fractions, air inclusions, and geometrical realizations following the virtual concrete laboratory approach of [19]. Quartzitic coarse aggregates are distributed randomly within the mortar matrix according to predefined volumetric grading curves, alongside explicit air inclusions representing pores and voids.

The simulation employs one ultrasonic source and one receiver, both positioned centrally in the x–y plane with a separation of 300mm along the *z*-axis. The source is modeled as a six-component moment tensor where only the xx and zz components are activated, reproducing the radiation pattern of transducers used in experimental studies [20]. The emitted signal is calibrated based on laboratory measurements with a central frequency of 60kHz [2]. At the receiver, the recorded signal corresponds to the mean value of the xx and zz stress tensor components. Wave propagation is solved using explicit finite-difference operators of second order in time and second order in space. A total of 80,000 time steps are computed with a fixed time increment $\Delta t=5\times 10^{-8}\;s$, resulting in a total simulated duration of 4ms.

Using the elastic parameters in Table 1, the shear-wave velocity in the mortar is $v_{s,\mathrm{mortar}}\approx 2070\;m/s$, and the maximum compressional-wave velocity in the aggregate phase is $v_{p,\mathrm{agg}}\approx 5720\;m/s$. At the source central frequency of 60kHz, the shear wavelength in the mortar is approximately $\lambda_{s}\approx 34.5\;\mathrm{mm}$, yielding approximately 69 grid points per wavelength for Δx=0.5mm. The numerical stability criterion is satisfied by the Courant number $C=v_{\max}\Delta t/\Delta x$, which evaluates to C≈0.572 using $v_{p,\mathrm{agg}}$.

Regarding numerical accuracy and sensitivity, while these discretization metrics confirm adequate sampling at the central frequency, a systematic grid-refinement study was not performed. Consequently, potential numerical dispersion, grid-induced anisotropy, and sensitivity to computational parameters across the full frequency spectrum of the source signal have not been formally bounded, and results are interpreted as simulations obtained at the specified resolution.

For signal processing, the cross-correlation threshold was set to 0.7, the dv/v search range to ±12%, and the search resolution to approximately 0.02 % (1201 trial values across the search interval). The coda time window started at time step 1000 (0.05ms) and ended at time step 10000 (0.5ms), unless stated otherwise.

The concrete model consists of three distinct material phases whose properties, adopted from [7], are summarized in Table 1.

### 3.2. Moisture Modelling

The relationship between moisture conditions and the quality factor *Q* is modeled following experimental observations by [8]. In concrete, relative humidity (RH) variations alter pore saturation, affecting anelastic energy dissipation without significantly changing bulk elastic stiffness under specific modeling assumptions.

In this study, a water-to-cement ratio (w/c) of 0.45 is considered. Moisture variations are simulated by adjusting relative humidity from 35% to 95%, corresponding to quality factor *Q* values summarized in Table 2.

Moisture is represented exclusively through changes in the quality factor *Q* (attenuation), while elastic moduli, density, wave speeds, and pore-fluid properties are held constant across relative humidity levels. Therefore, the following results on relative velocity applies strictly to this attenuation-based modelling assumption and does not capture moisture-induced changes in stiffness, swelling, or pore-pressure coupling in real concrete. The source and applicability of this relative-humidity-to-*Q* relationship derive directly from the experimental calibrations established by [8].

### 3.3. Temperature Modelling and Calibration

Temperature variations induce measurable velocity changes and waveform decorrelation in concrete. To model thermal effects, temperature changes are applied by scaling the elastic moduli of the mortar matrix via a temperature-dependent scaling factor $S_{t}$, which modifies compressional ($v_{p}$) and shear ($v_{s}$) wave velocities according to Equations (3) and (4):

(3) $$v_{p}=\sqrt{\frac{S_{t}E\left(1-\nu \right)}{\rho (1+\nu )(1-2\nu )}}$$

(4) $$v_{s}=\sqrt{\frac{S_{t}E}{2\rho (1+\nu )}}$$

The temperature range investigated spans from 0 °C to 40 °C (with reference temperature $T_{\mathrm{ref}}=20\;{}^{\circ}C$, where $S_{t}=1.000$). Table 3 reconciles the simulated temperature conditions, scaling factors $S_{t}$, and corresponding physical states.

The temperature-dependent scaling of the mortar matrix was calibrated against experimental trends reported by [8], distinguishing model calibration against known macroscopic slopes from independent numerical validation of the resulting wavefield responses.

The temperature-dependent scaling and calibration of the numerical model was applied exclusively to the mortar matrix in order to reflect the physical mechanisms governing ultrasonic wave propagation in concrete under environmental temperature variations. Experimental investigations based on coda wave interferometry have demonstrated that, when relative humidity is controlled and irreversible damage is avoided, ultrasonic relative velocity variations in concrete exhibit a stable and reproducible linear dependence on temperature [8]. Results evidence slopes ranging 0.037–0.052 dvv(%)/K for relative humidities varying between 35 % and 95 %. Although the experimental observations are made at the macroscopic scale, the interpretation of the measured d*v*/*v*–temperature relationship is consistent with the understanding that temperature primarily affects the elastic response of the cementitious matrix rather than that of the aggregates.

From a micromechanical perspective, the mortar matrix constitutes the continuous phase in concrete and governs the effective elastic moduli that control wave speeds. Theoretical and experimental studies on concrete mechanics have long established that the elastic properties of cement paste and mortar are more sensitive to temperature-induced changes than those of mineral aggregates, due to the presence of hydration products, pore fluid interactions, and thermally induced micro-prestress effects in the cementitious phase [21,22]. In contrast, aggregates generally behave as stiff elastic inclusions whose elastic moduli remain comparatively stable under moderate temperature variations typical of service conditions [23]. Reviews of ultrasonic and coda wave methods further emphasize that small reversible changes in elastic moduli within the load-bearing matrix phase dominate measured velocity variations in heterogeneous materials such as concrete [9].

Applying temperature-dependent scaling to both the mortar matrix and the aggregates would therefore distribute thermal sensitivity into phases that are not expected to contribute significantly to the experimentally observed d*v*/*v*–temperature relationship, leading to an artificial representation of the material response. By restricting temperature effects to the mortar matrix, the numerical model reproduces the experimentally observed linear velocity variation while preserving a physically meaningful separation between thermal effects and subsequent damage-induced stiffness degradation, which predominantly initiates and evolves within the cementitious phase.

### 3.4. Damage Modelling and Loading Protocol

Mechanical damage is simulated using the Discrete Element Method (DEM) to capture crack initiation, propagation, and microstructural degradation under uniaxial tension [2,7]. Tensile loading is applied because concrete is significantly weaker in tension than in compression (tensile strength is approximately 10% of compressive strength).

To resolve the mechanical response, the loading protocol comprises 18 total DEM loading steps as detailed in Table 4.

**Figure 4:**
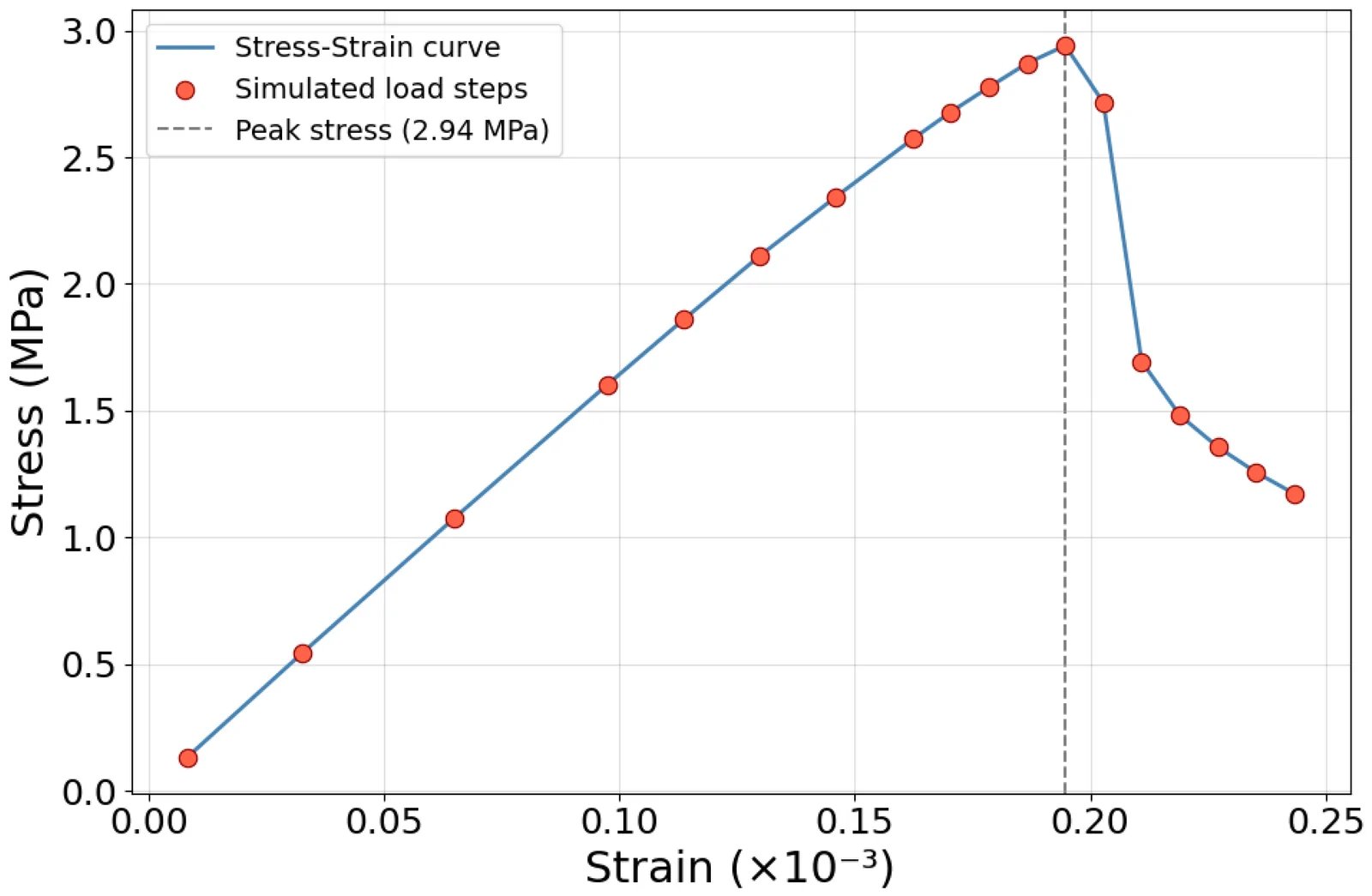
Stress–strain curve corresponding to each simulated load step.

**Figure 5:**
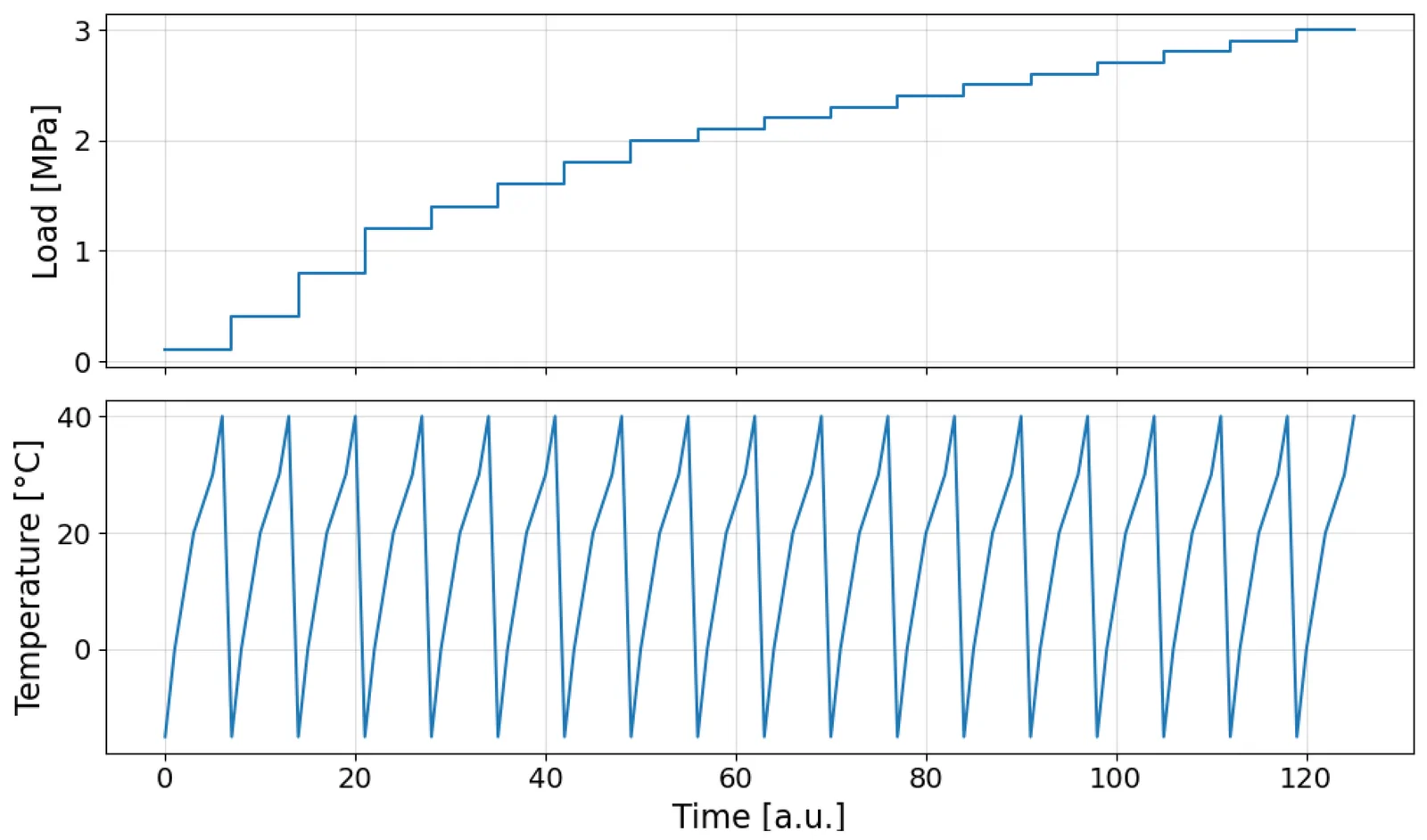
Applied mechanical load history (top) and temperature cycles (bottom) during simulations.

The scalar damage index *D* is defined within continuum damage mechanics as

(5) $$D=1-\frac{E}{E_{0}}$$

where *E* is the secant Young’s modulus at a given load step and $E_{0}$ is the reference modulus of the intact specimen at the initial step (0.1MPa). Stress-displacement data and derived secant moduli (Figure 4) govern the stiffness reduction. The damaged elastic and density fields from the DEM simulation are transferred directly into the finite-difference wave-propagation model at each selected step. Figure 5 illustrates the applied thermal and mechanical loading histories.

## 4. Results and Discussion

### 4.1. Impact of Moisture on the Coda Wave Signal

**Figure 6:**
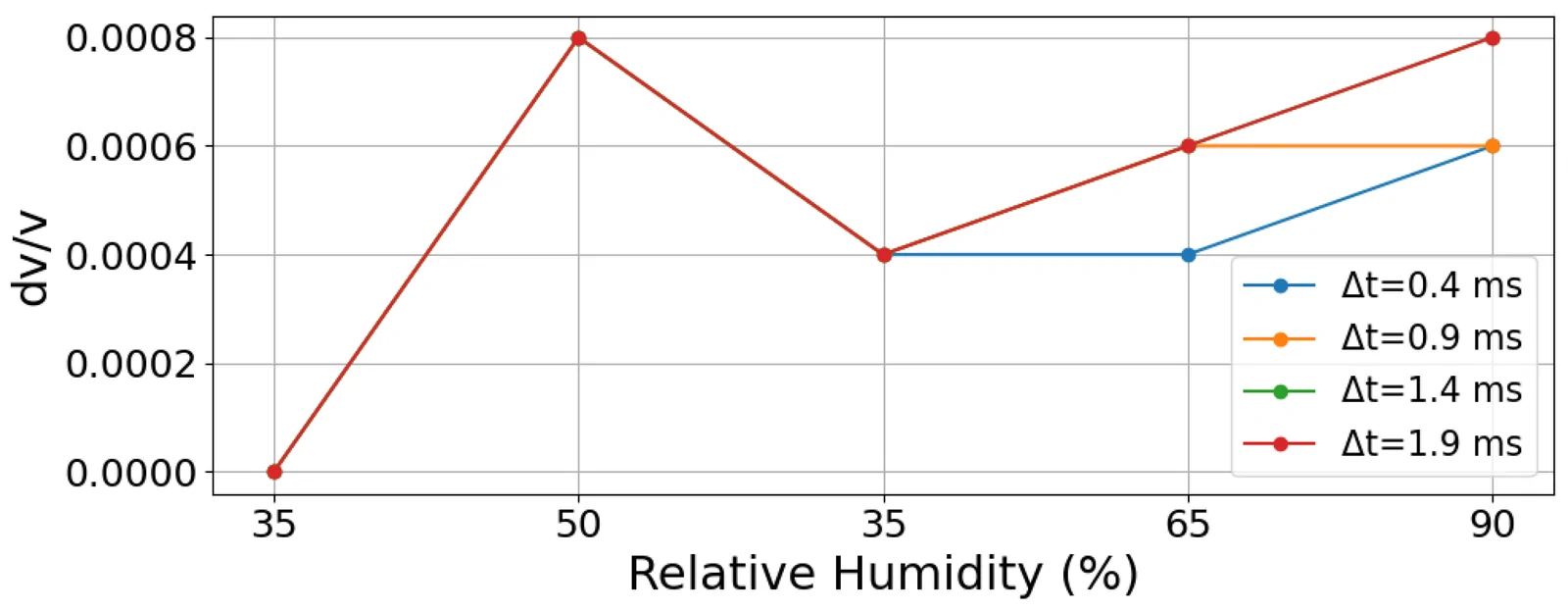
Relative velocity variation dv/v plotted against relative humidity for different time windows (reference start time 0ms).

To evaluate the effect of moisture changes on the coda wave signal, relative humidity variations (35% to 95% RH) were applied. Relative velocity changes (dv/v) and waveform decorrelation (1−CC) were evaluated across multiple time windows (Δt=0.4ms, 0.9ms, 1.4ms, 1.9ms). Figure 6 depicts dv/v versus relative humidity.

The results demonstrate a negligible effect of moisture on relative wave velocities, with maximum velocity changes remaining below 0.08% across the entire RH range. No clear deterministic trend is observed due to the extremely small magnitude of the velocity variations.

**Figure 7:**
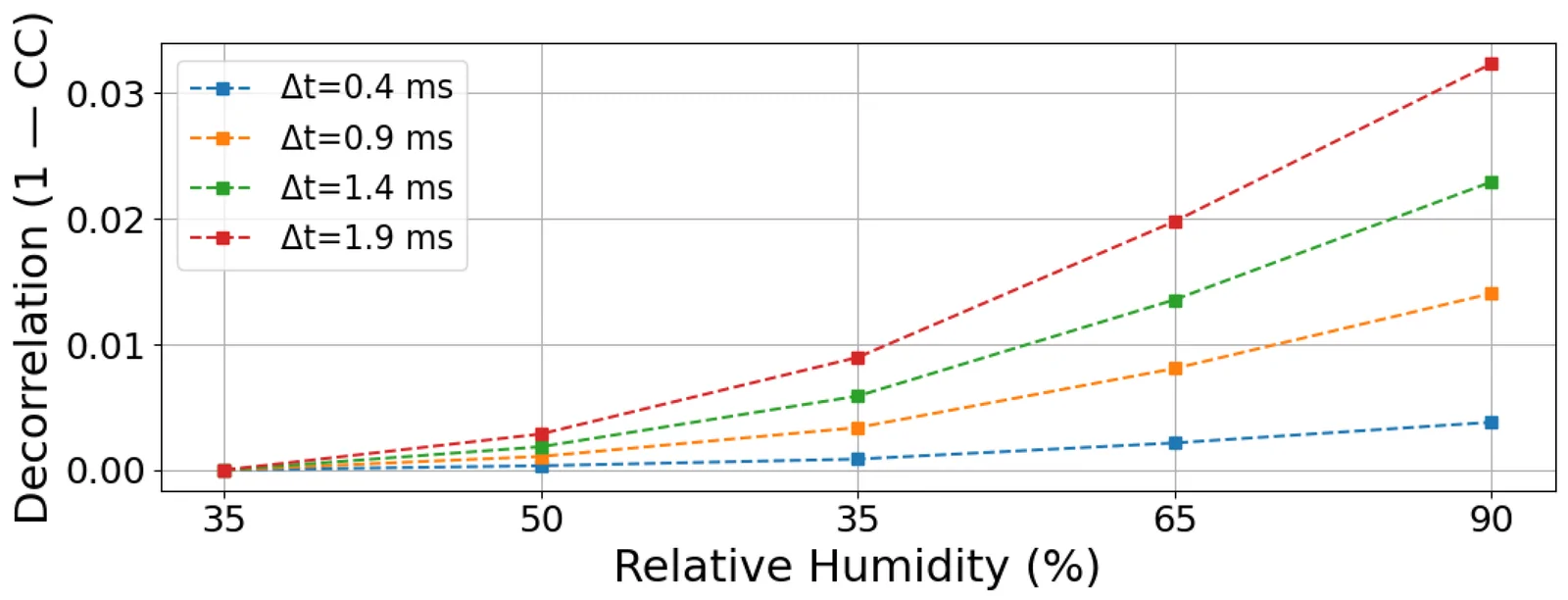
Decorrelation (1−CC) against relative humidity for different time windows.

Conversely, Figure 7 illustrates that waveform decorrelation (1−CC) increases systematically with relative humidity and longer time windows. This behavior confirms that increased RH enhances attenuation (1/Q), weakening coherent signal phases and increasing relative noise contribution. However, when signal amplitude and signal-to-noise ratio (SNR) are strictly controlled, decorrelation correlates directly with attenuation changes rather than velocity shifts.

### 4.2. Temperature and Combined Effect of Temperature–Moisture on the Coda Wave

**Figure 8:**
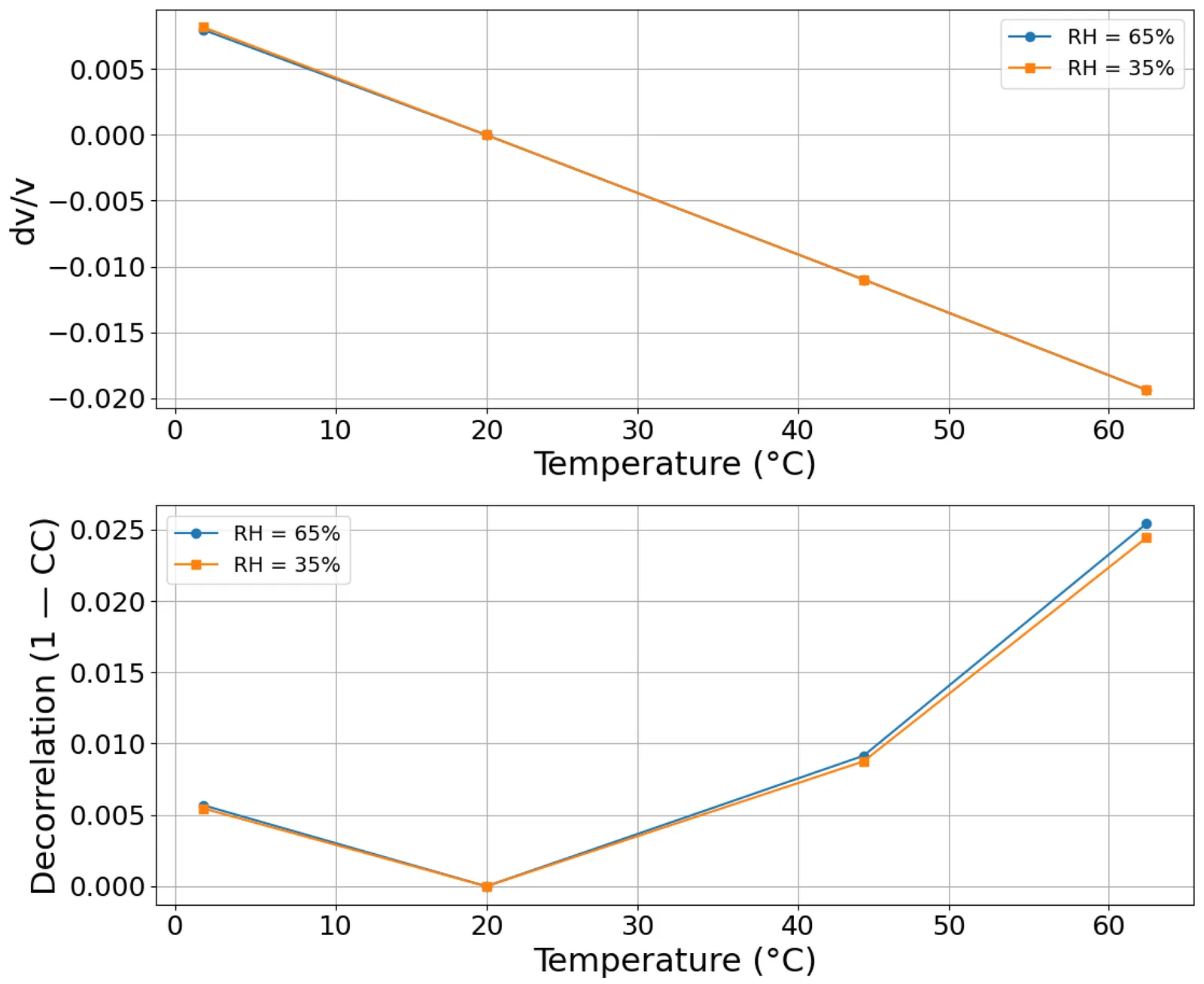
Relative velocity and decorrelation against temperature applied to mortar only for 35% and 65% RH.

To evaluate thermal and combined environmental effects, temperature variations were applied to the mortar matrix at two distinct relative humidity levels (65% RH, Q=180; and 35% RH, Q=285). Figure 8 presents dv/v and decorrelation against the thermal scaling factor and temperature.

The relative velocity variation exhibits a clear, highly linear dependence on temperature, decreasing proportionally with increasing temperature. Specifically, decreasing temperature from 20 °C to 2 °C increases dv/v by 0.5% to 1%, whereas increasing temperature from 20 °C to 55 °C decreases dv/v by approximately 2%. Furthermore, the linear trends for 35% and 65% RH perfectly overlap, confirming that moisture variations have an inconsequential effect on velocity changes regardless of temperature.

**Figure 9:**
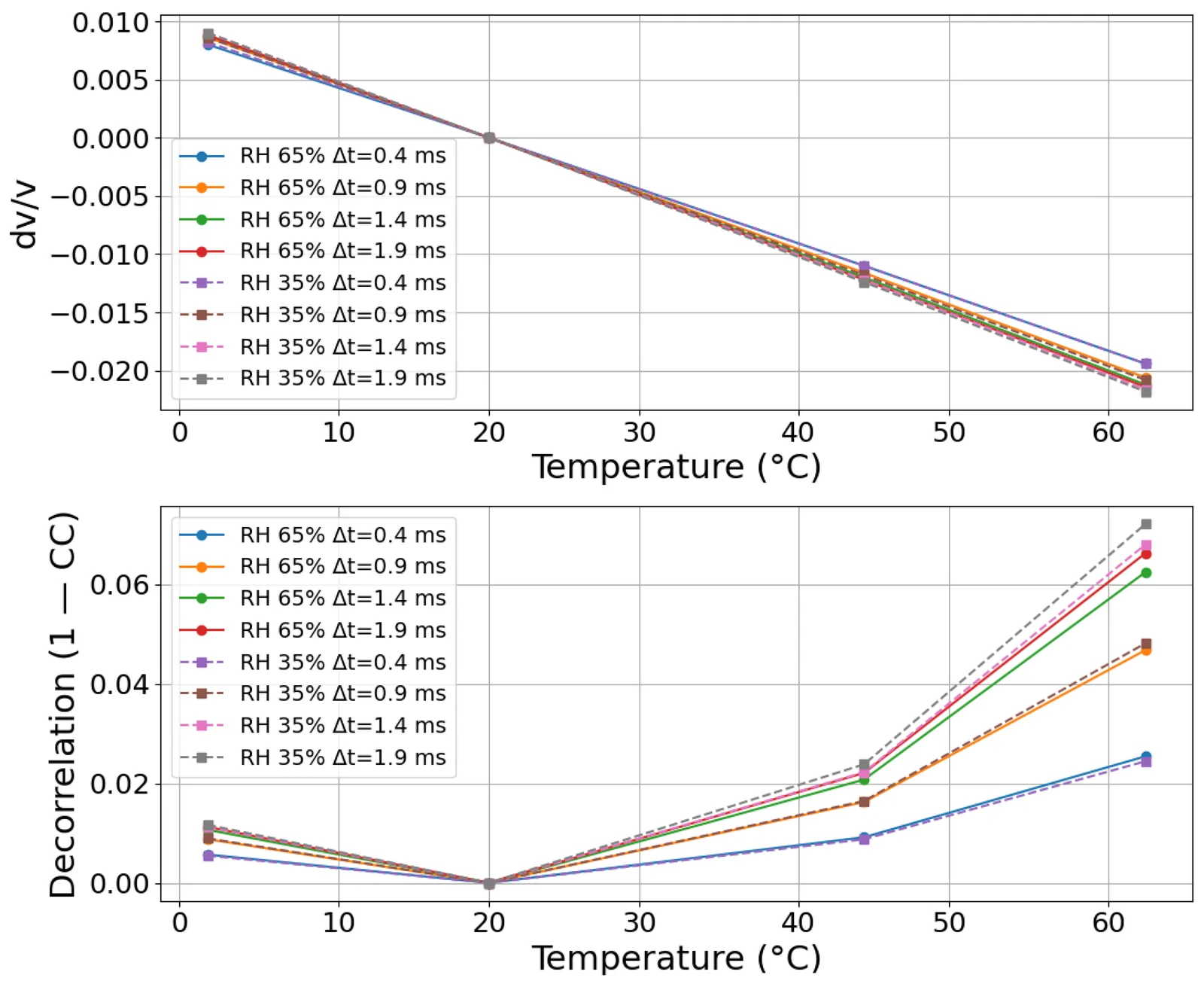
Relative velocity and decorrelation against temperature applied to mortar only across different time windows.

Decorrelation exhibits a parabolic increase at elevated or reduced temperatures, reaching up to 0.06 at 55 °C (Figure 9). Figure 9 further confirms that time window selection affects decorrelation magnitude but preserves the underlying trends.

### 4.3. Effect of Damage and Temperature on the Coda Signal

**Figure 10:**
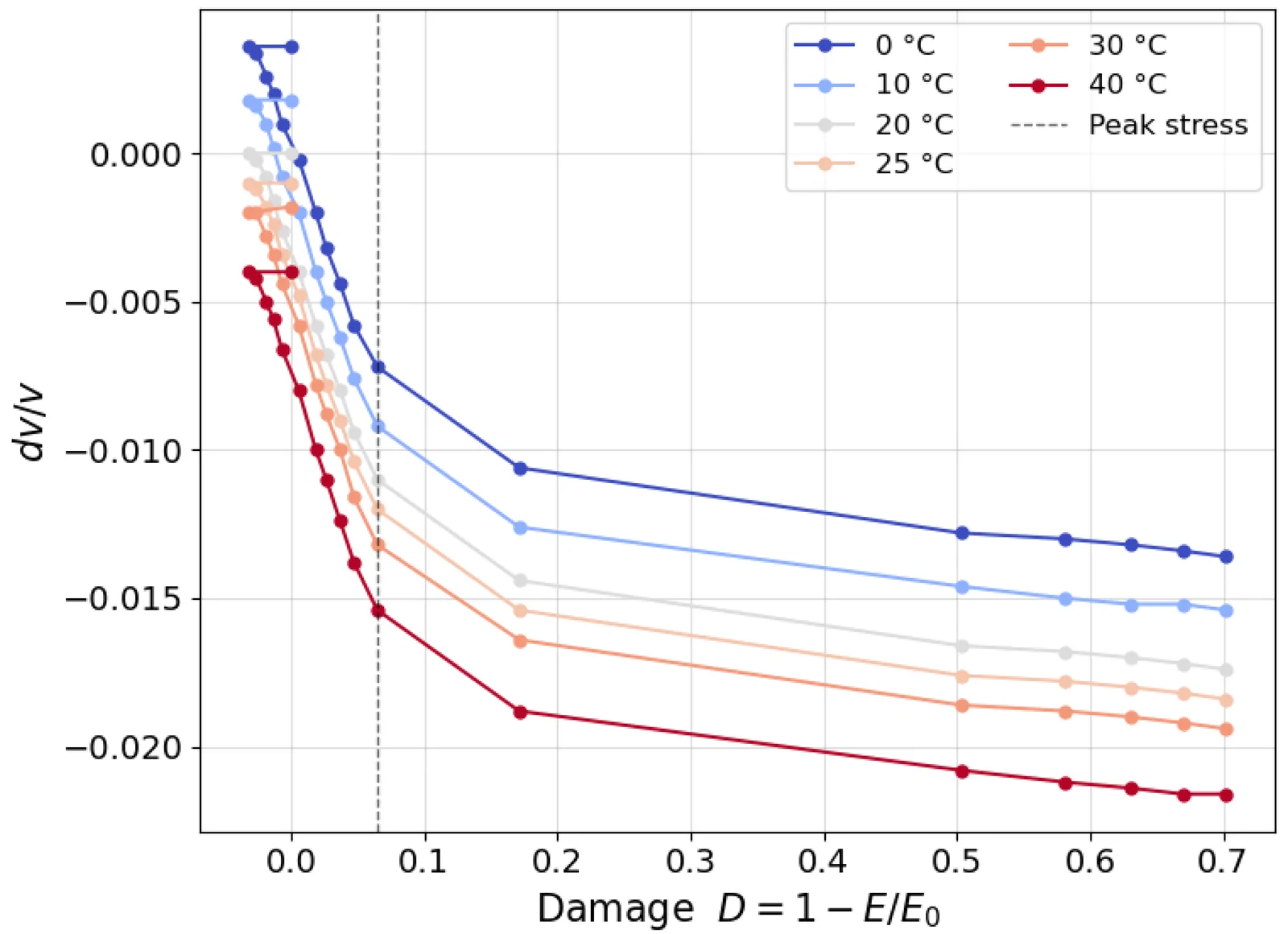
Relative velocity dv/v against damage index *D* for multiple temperature levels.

The combined effect of mechanical damage and temperature on dv/v is illustrated in Figure 10. Relative velocity variations reach approximately −1.7% at extreme load steps.

**Figure 11:**
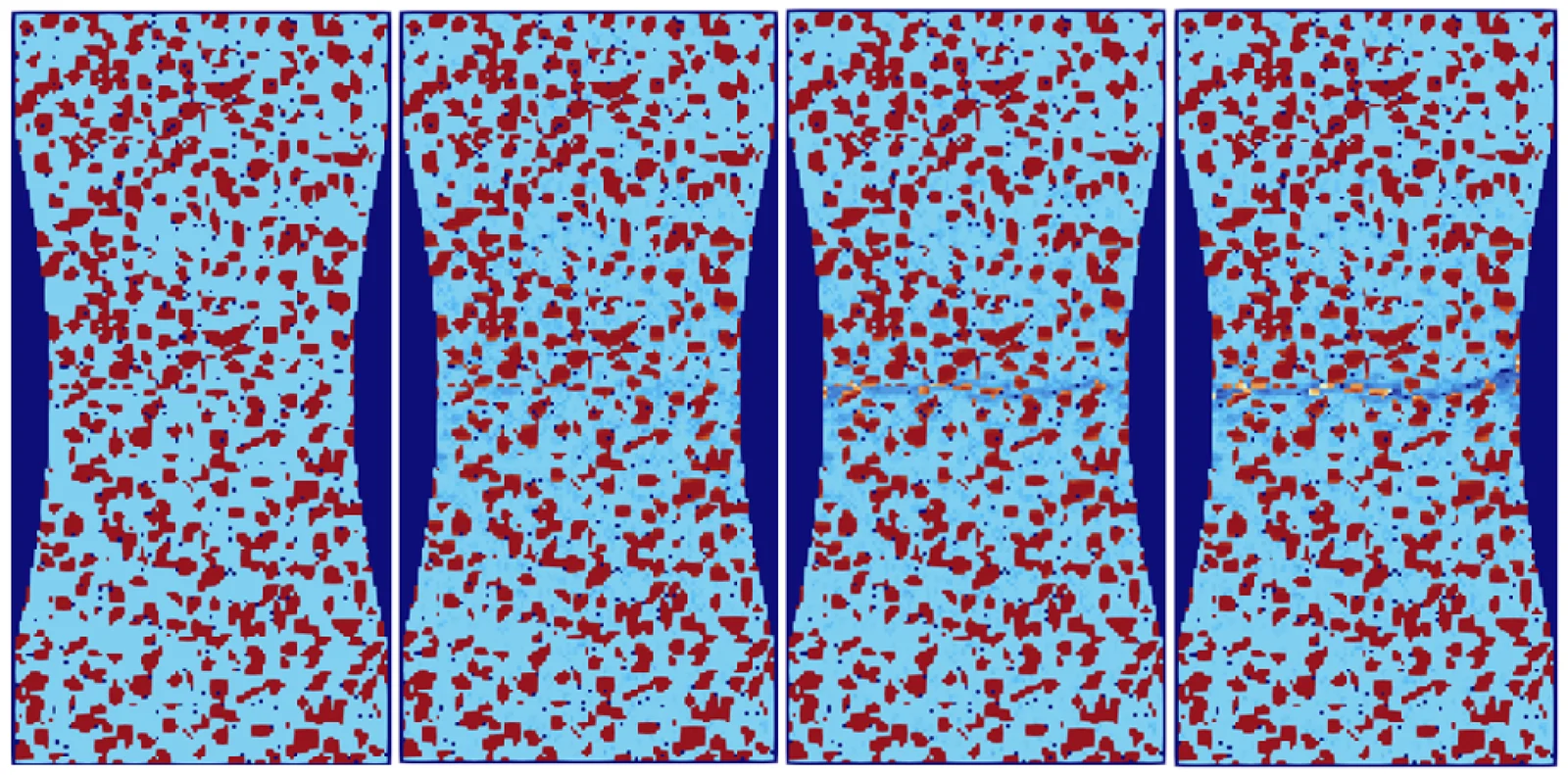
Evolution of crack formation in the density field during increasing tension (left to right: 0.1MPa, 2.4MPa, 2.5MPa, 3.0MPa).

Based on the Young’s modulus evolution and crack density fields (Figure 11), three distinct deformation regimes are identified:

1. *Stable microcracking (D<0.08, load steps 1–24):* Gradual stiffness degradation as tensile microcracks nucleate and propagate uniformly up to peak stress ($\sigma_{\mathrm{peak}}\approx 2.94\;\mathrm{MPa}$ at step 24) [2]. This pre-peak regime corresponds to the range in which diffuse damage distributes across the specimen volume before localization, a behavior widely documented for quasi-brittle materials under both compression [7] and tension [2]. The coda wavefield is particularly sensitive in this regime because the multiply scattered waves sample the entire evolving microcrack network [5,9].
2. *Post-peak onset (0.08≤D<0.2, load step 25):* Sharp stress drop and stiffness degradation from D≈0.065 to 0.171, manifesting as a steep drop in dv/v.
3. *Post-peak softening regime (D>0.2, load steps 26–30):* Residual load transfer via aggregate interlock and friction along open macrocracks. Stiffness decreases at a reduced rate governed by crack opening displacement.

#### Thermal Correction and Damage Isolation

To isolate mechanical damage from thermal effects, a linear thermal correction was applied. Equation (6) defines the correction using a single model-specific calibration coefficient $\alpha_{\mathrm{sim}}=-1.883\times 10^{-4}\;{}^{\circ}C^{-1}$ (representing a sensitivity magnitude of 0.0188%/K), derived from the reference state ($T_{\mathrm{ref}}=20\;{}^{\circ}C$) and first load step across the six simulated temperatures:

(6) $${\left(dv/v\right)}_{\mathrm{corr}}={\left(dv/v\right)}_{\mathrm{raw}}-\alpha_{\mathrm{sim}}\left(T-T_{\mathrm{ref}}\right)$$

**Figure 12:**
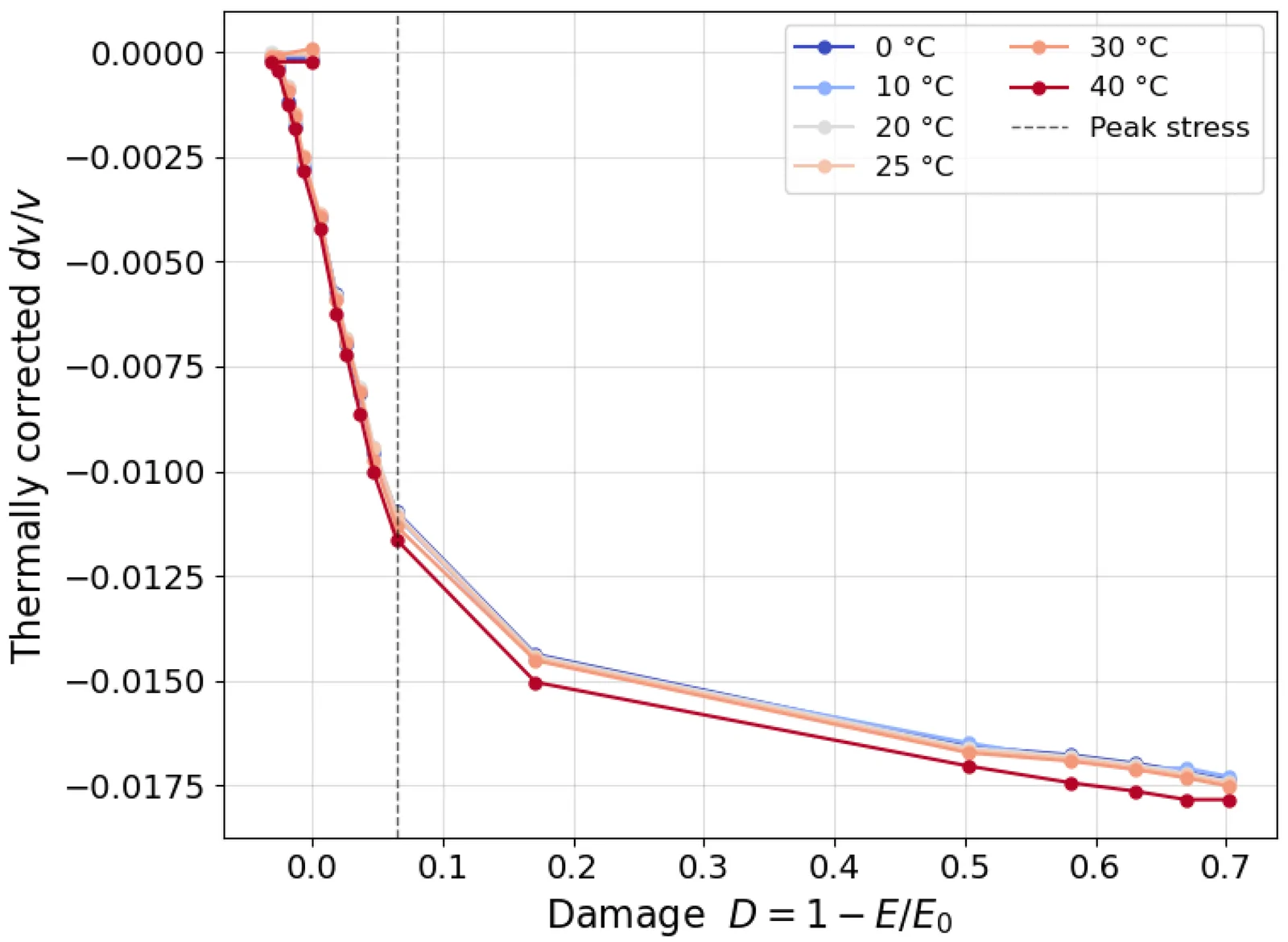
Thermally corrected relative velocity change dv/v against damage index *D*.

Figure 12 displays the thermally corrected dv/v versus damage index *D*.

The thermal correction substantially reduces, but does not completely eliminate, the temperature dependence. Across the 18 selected load states, the inter-temperature range (max − min dv/v) decreases from a mean of 0.79% before correction to 0.051% after correction (a 93.6% reduction). The residual temperature dependence after correction ranges from 0.032% to 0.075% across sampled load states (0.067% at peak stress). This residual dependence is explained by temperature-damage interactions and changes in effective elastic state not captured by a single offset coefficient calibrated at the initial state.

## 5. Conclusions

This study investigated the sensitivity of CWI to mechanical load, temperature, and moisture in concrete through a fully numerical framework. The results confirm that each parameter acts through distinct mechanisms: temperature produces global velocity shifts via thermoelastic mortar softening; moisture alters attenuation globally via *Q* variations with negligible velocity effects; and mechanical damage induces local, nonlinear stiffness changes.

Key findings and limitations restricted to the investigated numerical configuration include:

- Moisture variations modeled via attenuation (*Q*) alone produce negligible velocity shifts (<0.08%), suggesting that dedicated moisture monitoring may not be strictly necessary for velocity-based indicators under these specific assumptions.
- Temperature variations produce velocity shifts of the same magnitude as mechanical damage, confirming that thermal compensation is essential for field CWI campaigns. Standard linear thermal correction reduces inter-temperature variation by 93.6%.
- Damage analysis identified three regimes (diffuse microcracking, post-peak onset, and softening), highlighting CWI’s sensitivity to early-stage degradation.
- Practical recommendations regarding early-warning applications and field-monitoring capability require further validation against measurement noise, detection thresholds, environmental variability, and multiaxial loading.

## Figures and Tables

**Table 1:** Elastic parameters of the three different phases of the concrete model (*E*: Young’s modulus; ν: Poisson’s ratio; ρ: density) [7].

Phase	*E* (GPa)	ν	ρ (kg/m^3^)
Aggregates	84.6	0.12	2670
Mortar	21.5	0.19	2105
Air inclusions	0.0	–	0.0001

**Table 2:** Correspondence between relative humidity (RH) and quality factor *Q* [8].

RH (%)	*Q*
35	285
50	215
65	180
80	150
95	130

**Table 3:** Reconciled correspondence between temperature *T* and thermal scaling factor $S_{t}$.

Temperature *T* (°C)	Scaling Factor $S_{t}$
0	1.033
10	1.016
20	1.000
25	0.992
30	0.984
40	0.967

**Table 4:** Selected DEM load steps and corresponding stress levels used for wave-propagation simulations.

DEM Load Step	Applied Load (MPa)
1	0.1
4	0.4
8	0.8
12	1.2
14	1.4
16	1.6
18	1.8
20	2.0
21	2.1
22	2.2
23	2.3
24	2.4
25	2.5
26	2.6
27	2.7
28	2.8
29	2.9
30	3.0

## Data Availability

The data are available upon request from the corresponding authors.
